# Oral Bioavailability of Omega-3 Fatty Acids and Carotenoids from the Microalgae *Phaeodactylum tricornutum* in Healthy Young Adults

**DOI:** 10.3390/md19120700

**Published:** 2021-12-10

**Authors:** Lena Stiefvatter, Katja Lehnert, Konstantin Frick, Alexander Montoya-Arroyo, Jan Frank, Walter Vetter, Ulrike Schmid-Staiger, Stephan C. Bischoff

**Affiliations:** 1 Institute of Nutritional Medicine, University of Hohenheim, Fruwirthstr. 12, 70593 Stuttgart, Germany; Lena.stiefvatter@uni-hohenheim.de; 2 Institute of Food Chemistry, University of Hohenheim, 70593 Stuttgart, Germany; Katja.Lehnert@uni-hohenheim.de (K.L.); Walter.Vetter@uni-hohenheim.de (W.V.); 3 Institute of Interfacial Process Engineering and Plasma Technology, University of Stuttgart, 70569 Stuttgart, Germany; Konstantin.Frick@igb.fraunhofer.de; 4 Department of Food Biofunctionality, Institute of Nutritional Sciences, University of Hohenheim, 70593 Stuttgart, Germany; alexander.montoya@nutres.de (A.M.-A.); jan.frank@uni-hohenheim.de (J.F.); 5 Fraunhofer Institute for Interfacial Engineering and Biotechnology IGB, Innovation Field Algae Biotechnology-Development, 70569 Stuttgart, Germany; ulrike.schmid-staiger@igb.fraunhofer.de

**Keywords:** microalgae, bioavailability, fatty acids, omega-3 fatty acids, eicosapentaenoic acid, fucoxanthin

## Abstract

The microalgae *Phaeodactylum tricornutum* (PT) contains valuable nutrients such as proteins, polyunsaturated omega-3 fatty acids (*n*-3 PUFA), particularly eicosapentaenoic acid (EPA) and some docosahexaenoic acid (DHA), carotenoids such as fucoxanthin (FX), and beta-glucans, which may confer health benefits. In a randomized intervention trial involving 22 healthy individuals, we administered for two weeks in a crossover manner the whole biomass of PT (5.3 g/day), or fish oil (FO) containing equal amounts of EPA and DHA (together 300 mg/day). In an additional experiment, sea fish at 185 g/week resulting in a similar EPA and DHA intake was administered in nine individuals. We determined the bioavailability of fatty acids and carotenoids and assessed safety parameters. The intake of PT resulted in a similar increase in the *n*-3 PUFA and EPA content and a decrease in the PUFA *n*-6:*n*-3 ratio in plasma. PT intake caused an uptake of FX that is metabolized to fucoxanthinol (FXOH) and amarouciaxanthin A (AxA). No relevant adverse effects occurred following PT consumption. The study shows that PT is a safe and effective source of EPA and FX—and likely other nutrients—and therefore should be considered as a future sustainable food item.

## 1. Introduction

The world population is growing, while food sources are limited and become eventually further limited because of climate changes, resulting in serious restrictions and a need for novel food sources [1]. Nutritional protein from meat and fish will be limited in the future, and microalgae have been proposed as a possible and sustainable alternative protein source. Microalgae can be harvested from the oceans but also grown in open ponds or photobioreactors [2] and thus, no farmland is needed to grow them. The composition of microalgae varies and includes—besides proteins—also lipids, especially *n*-3 PUFA, and antioxidants (e.g., carotenoids and vitamin E), which have potential physiological and health-beneficial effects in humans [3,4,5]. Carotenoids, which cannot be synthesized by humans and animals, are of particular interest here, because they must be ingested through food or supplements [6]. Relevant sources for carotenoids are egg yolks since animals accumulate lutein and zeaxanthin there [7], milk, salmon, fish, or crustaceans, and in the future possibly also selected microalgae [8]. A prominent carotenoid, β-carotene, is an antioxidant, thus protecting from reactive oxygen species (ROS) and free radical-induced damage, and a precursor of vitamin A [9,10]. Xanthophylls, another type of carotenoids, exhibit higher antioxidant capacity compared to β-carotene by scavenging free radicals and quenching singlet oxygen [11]. Fucoxanthin (FX), is the major xanthophyll in brown-colored micro-and macroalgae (seaweeds) [12,13]. FX acts as an antioxidant, causes a reduction of plasma and liver triglycerides, and has a positive effect on cholesterol regulating enzymes in preclinical studies [14]. The brown color comes from the combination of the green chlorophyll and the red xanthophyll FX.

Microalgae are primary producers of *n*-3 PUFA, especially eicosapentaenoic acid (EPA) and docosahexaenoic acid (DHA), which are essential for human nutrition because they cannot be synthesized in the human body [4]. Microalgae are not currently used as a primary food source of *n*-3 PUFA, even though they are at the beginning of the food chain, since fish, our major *n*-3 PUFA source, take the fatty acids up from microalgae. Since microalgae provide both *n*-3 PUFA and protein, they could replace to some extent fish as recommended food. This is of particular interest since the demand for EPA and DHA continues to rise and at the moment there is a global gap of 1.1 million tons, as fish currently accounts for only 15% and krill for 0.3% of the calculated amounts needed by humans daily [15].

Precursors such as alpha-linolenic acid (ALA) are found e.g., in green leafy vegetables, nuts, and flaxseed, but they cannot be converted to EPA and DHA in the body at high rates [16]. For a healthy diet, eating fish for 1–2 servings per week is recommended by the DGE (German Nutrition Society) and EFSA (European Food Safety Authority) to ensure a daily intake of 250 mg EPA + DHA [17], to improve markers or risk factors associated with cardiovascular diseases [18]. If the world population would follow these recommendations, our waters would be fished dry.

The amount of EPA and DHA is varying depending on fish species and farming conditions. For supplementation of EPA + DHA, some people consume fish oil (FO) capsules. However, FO capsules are not sustainable either, since about 20–25 kg of fish is needed to produce 1 kg FO [19] and two-third of the current FO production is used for fish feed [20]. In this context, EPA- and DHA-rich microalgae represent an innovative food source capable of at least partially replacing fish and FO. Prominent *n*-3 PUFA producers are *Phaeodactylum tricornutum* (PT), *Nannochloropsis*, *Thraustochytrium*, *Ulkenia*, *Schizochytrium*, and *Crypthecodinium cohnii* sp., of which the last three in the list have been classified as oil as generally recognized as safe (GRAS) [21]. *Odontella aurita* as a whole alga, *Ulkenia* sp.-oil, and *Schizochytrium* sp.-oil are approved under the EU Commission [22].

The microalgae PT, which belongs to the diatom family, has received attention in recent years because it is a particularly rich source of EPA [23,24]. In addition to proteins and *n*-3 PUFA, PT contains large amounts of β-carotene and FX [25]. PT is not yet approved as whole biomass, yet an EPA-rich oil made of PT is on the market in the U.S.A [26] and a 2% FX-rich extract of PT has GRAS status (“BrainPhyt-PhaeoSOL”) [27]. We have previously shown a good bioavailability of fatty acids from microalgae in preclinical studies [28,29,30]. 

This study demonstrates the bioavailability of fatty acids, carotenoids, and vitamins from PT and the safety of ingestion of the microalgae as whole biomass in humans. In a randomized intervention trial involving healthy individuals, the whole biomass of PT, or commercial FO capsules containing equal amounts of EPA + DHA were administered for two weeks in a cross-over manner. For a detailed safety assessment, intestinal health was investigated during the study by measuring intestinal barrier markers, as well as microbiota composition and function. In a subset of study participants, PT effects were also compared with eating fish providing similar amounts of EPA + DHA as the study products.

## 2. Results

### 2.1. Adverse Effects during the Intervention

PT dissolved in water after ball milling was generally well accepted by the study participants, although taste could be improved. Adverse effects following administration of PT and other study products were monitored by a diary, in which the participants documented their complaints, and by a questionnaire conducted at each study visit by the personnel. No serious side effects were reported, neither by the study participants in their diaries nor by the personnel in the questionnaires (Table 1). In detail, 15 participants did not mention any adverse events while taking PT or FO and no one had a side effect from eating fish. Most side effects occurred to a minimal or mild extent after PT consumption, e.g., headache, and feeling of thirst, skin problems, and reduced appetite. Gastrointestinal problems such as bloating were described for both treatments, PT and FO. Other side effects were reported occasionally, such as flatulence, stomach pain, constipation, and diarrhea. 

Adverse effects were documented in a diary by the participants and recorded upon questionnaire by the study personnel at the visits. Values from completers are expressed as absolute numbers. Minimal, transient symptoms with no impairment of the patient’s daily activities; Mild, consistent symptoms with moderate impairment of the patient’s daily activities; Severe, significant impairment of the patient’s daily activities. Abbreviations: PT, *Phaeodactylum tricornutum*; FO, fish oil.

### 2.2. Laboratory Parameters

**Figure 1:**
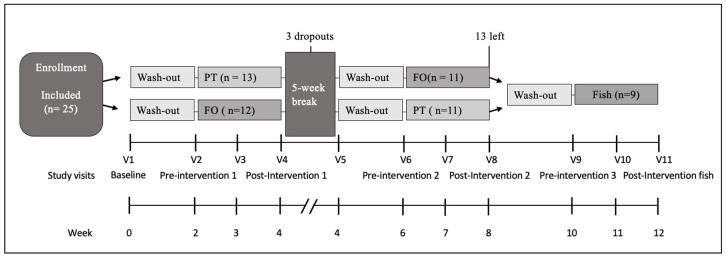
Study flow diagram. A total of 25 participants were randomized into two groups at V1 (baseline). The first and second part of the study (PT (and FO exposure, cross-over design) was completed by 22 individuals and were included for analysis. Of them, nine individuals volunteered to participate in the third part (fish exposure). Abbreviations: PT, *Phaeodactylum tricornutum*; FO, fish oil. For further details see text.

Different laboratory parameters were determined at 11 time points (V1–V11, see Figure 1 to examine the safety of PT consumption compared to FO and fish consumption (Table 2). During the PT intervention, uric acid concentrations increased from pre- to post-intervention (*p* = 0.004) but never differed to the baseline. In addition, PT intake was associated with an increase in high-density lipoprotein (HDL)-cholesterol (Chol) compared to the baseline (*p* = 0.01). Comparing laboratory parameters after the different interventions, we found differences between PTpost and FOpost for Chol (*p* = 0.003), HDL cholesterol (*p* = 0.003), low density lipoprotein (LDL) Chol (*p* = 0.003), as well triacylglycerols (TAG) (*p* = 0.009). As well, the LDL/HDL ratio was higher at PTpost compared to FOpost (*p* = 0.04). There was no significant difference between PT and fish intervention.

### 2.3. Change of Plasma Fatty Acids upon Intervention

**Figure 2:**
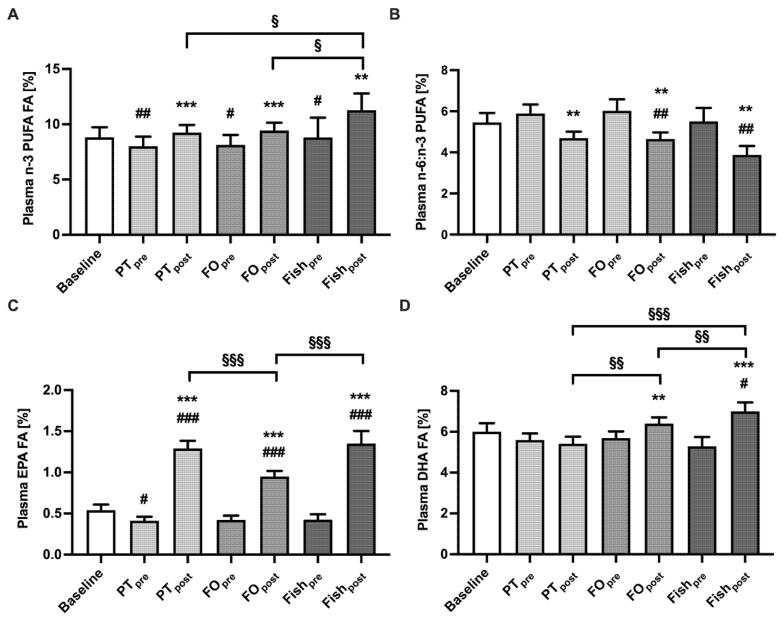
Change of plasma fatty acids concentrations upon intervention. *n*-3 PUFA (panel (**A**)), *n*-6 PUFA to *n*-3 PUFA ratio (**B**), EPA (**C**), and DHA (**D**) in plasma were determined before (“pre”) and after two weeks (“post”) intervention. Values are expressed in percent as mean ± SEM from 22 (PT, FO), or nine (Fish) participants. Statistics: * indicate differences to “pre”, # indicate differences to baseline, § indicate differences between different interventions. **/^##^/^§§^ *p* < 0.01, ***/^###^/^§§§^ *p* < 0.001. Abbreviations: PT, Interventions with *Phaeodactylum tricornutum*; FO, Intervention with fish oil; Fish, Intervention with salmon; *n*-3 PUFA, polyunsaturated omega-3 fatty acids; EPA, Eicosapentaenoic acid; DHA, Docosahexaenoic acid.

In order to determine the bioavailability of *n*-3 PUFA plasma fatty acids were measured for each intervention at baseline as well as pre- and post-intervention (Figure 2, and Supplementary Table S2). The *n*-3 PUFA plasma concentrations always decreased during the washout period from baseline to pre-intervention for all interventions (PT, *p* = 0.003; FO, *p* = 0.02; fish, *p* = 0.04), confirming that no *n*-3 PUFA was consumed at that time (Figure 2A). All *n*-3 PUFA-rich interventions increased plasma *n*-3 PUFA levels from pre- to post-intervention (PT, FO, *p* < 0.001; fish, *p* = 0.008), the increase being maximal after fish consumption (Fishpost compared to PTpost, *p* = 0.03; and Fishpost compared to FOpost, *p* = 0.03). The higher daily *n*-3 PUFA concentration of fish (554 mg/day) increased *n*-3 PUFA plasma concentrations from 8.8% to 11.3% (Δ2.5%) over two weeks. The increase was less pronounced for PT 8.0% to 9.25% (Δ1.24%) and FO 8.1% to 9.4% (Δ1.29%).

Our data show that abstaining from *n*-3 PUFA-rich foods increased the PUFA *n*-6:*n*-3 ratio from baseline to PTpre (*p* = 0.051), FOpre (*p* = 0.007) and Fishpre (*p* = 0.002) groups, but following supplementation with PT, FO or fish resulted in a decrease of the ratio after two weeks (PT, *p* = 0.001; FO, *p* = 0.005; fish, *p* = 0.002). The washout phase resulted in a decrease of EPA from baseline to all interventions but only significant to PTpre (*p* = 0.02). After a two-week consumption of the study products, all plasma EPA concentrations increased from baseline to post-intervention (*p* < 0.001) and from pre- to post-intervention (*p* < 0.001). The value of FOpost was lower compared to PTpost (*p* < 0.001) and the Fishpost was higher compared to FOpost (*p* = 0.03). PT increased EPA plasma concentrations from 0.4% to 1.3%, (Δ0.9%) higher compared to FO increase by 0.4% to 1.0% (Δ0.5%, *p* = 0.04). Fish increased EPA in plasma from 0.4% to 1.4% (Δ0.9%) comparable to PT. Since PT is not a DHA supplier, only the FO and Fish interventions increased DHA from pre- to post-intervention (FO, *p* = 0.004; fish, *p* = 0.0006) and Fish from baseline to Fishpost (*p* = 0.02). This results in higher DHA plasma concentrations for FOpost (*p* = 0.008) and Fishpost (*p* < 0.001) compared to PTpost. For further results see Supplementary Table S2.

### 2.4. Carotenoids and Tocopherol Concentrations before and after Intervention with PT

**Figure 3:**
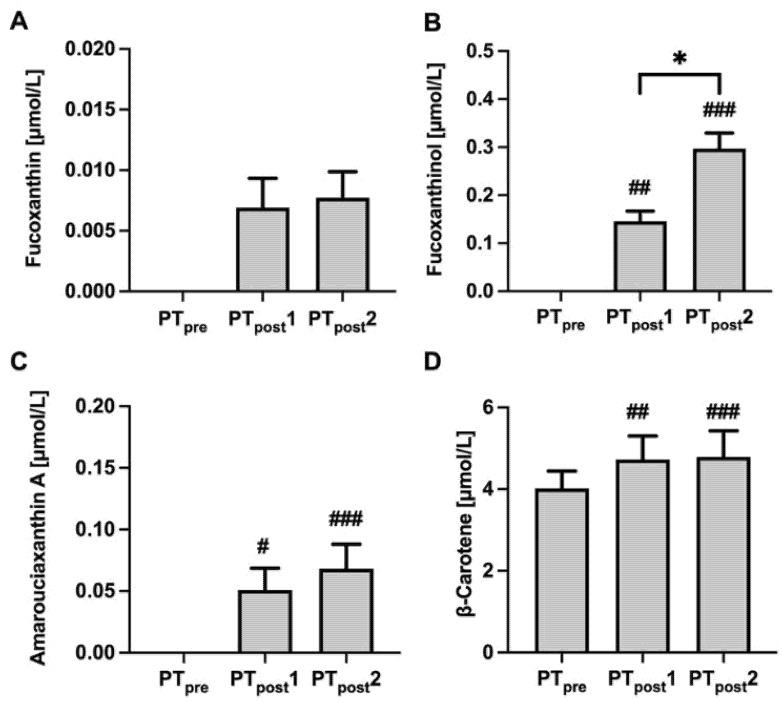
Plasma fucoxanthin (FX) and its metabolites as well as β-carotene concentrations before and after interventions with the microalgae *Phaeodactylum tricornutum* (PT). Plasma values of FX (**A**), fucoxanthinol (FXOH) (**B**), amarouciaxanthin (AxA) (**C**), and β-carotene (**D**) before intervention (PTpre) and one and two weeks after intervention (PTpost1, PTpost2) are shown as mean ± SEM from 22 participants. Statistics: # indicate difference to PTpre; * indicate difference between PTpost1 and PTpost2 */# *p* < 0.5, ## *p* < 0.01; ### *p* < 0.001.

The carotenoids FX and β-carotene as well as other carotenoids were measured within the PT intervention at three time points after pre-intervention (PTpre), post-intervention one week (PTpost1), and post-intervention two weeks (PTpost2). FX was detected in rather small amounts in plasma after one week and two weeks of PT ingestion (Figure 3A). FX metabolites could be detected at higher concentrations and more consistently in the study participants. FXOH, to which FX is hydrolyzed by cholesterol esterase in the intestinal tract, was detected after one week of exposure to PT, and further increased after two weeks of exposure (Figure 3B). AxA, to which FXOH is converted in the liver [31] and which accumulates mainly in adipose tissue, could be also detected after one and two weeks of PT consumption (Figure 3C).

Plasma β-carotene also increased after one and two weeks of PT consumption (Figure 3D). Due to the pro-vitamin A activity of β-carotene, we also measured retinol before and after PT exposure, but no increase was observed (data not shown). Other carotenoids, such as lutein/zeaxanthin, lycopene, β-cryptoxanthin, and α-carotene were detectable in plasma, but plasma concentrations did not change following PT consumption. Furthermore, plasma tocopherols did not change after two weeks of PT consumption (data not shown).

### 2.5. Gut Barrier Function and Gut Microbiome

**Figure 4:**
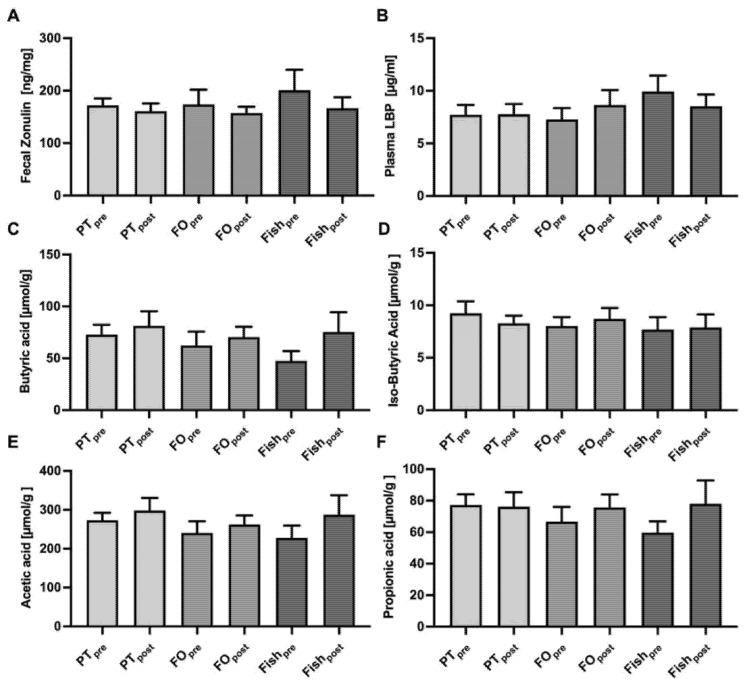
Assessment of gut barrier function and fecal short-chain fatty acid (SCFA) levels. Gut barrier function was analyzed by the validated biomarkers fecal zonulin (**A**), and plasma lipopolysaccharide-binding protein (LBP) (**B**). The SCFA butyric acid (**C**), iso-butyric acid (**D**), acetic acid (**E**), and propionic acid (**F**) were measured in feces before (“pre”) and after (“post”) intervention. Means ± SEM from 22 (PT, FO), or 9 (Fish) participants are shown. Abbreviations: see Figure 1.

Fecal zonulin and plasma LBP, two recently validated markers for intestinal permeability [32], were examined after consumption of PT, FO, and fish. Neither the fecal zonulin nor the plasma LBP were negatively affected by PT consumption or any of the other challenges. All values were above the normal ranges for fecal zonulin (61 ng/mg ± 46 ng/mL) or even higher in the healthy individuals who participated in the study. Plasma LBP concentrations were within the normal range (5–10 μg/mL) according to the kit manufactures information (Figure 4A,B). Measurement of the SCFA butyrate, iso-butyrate, acetate, and propionate in feces revealed no change following the consumption of the *n*-3 PUFA-rich study products (Figure 4C–F).

**Figure 5:**
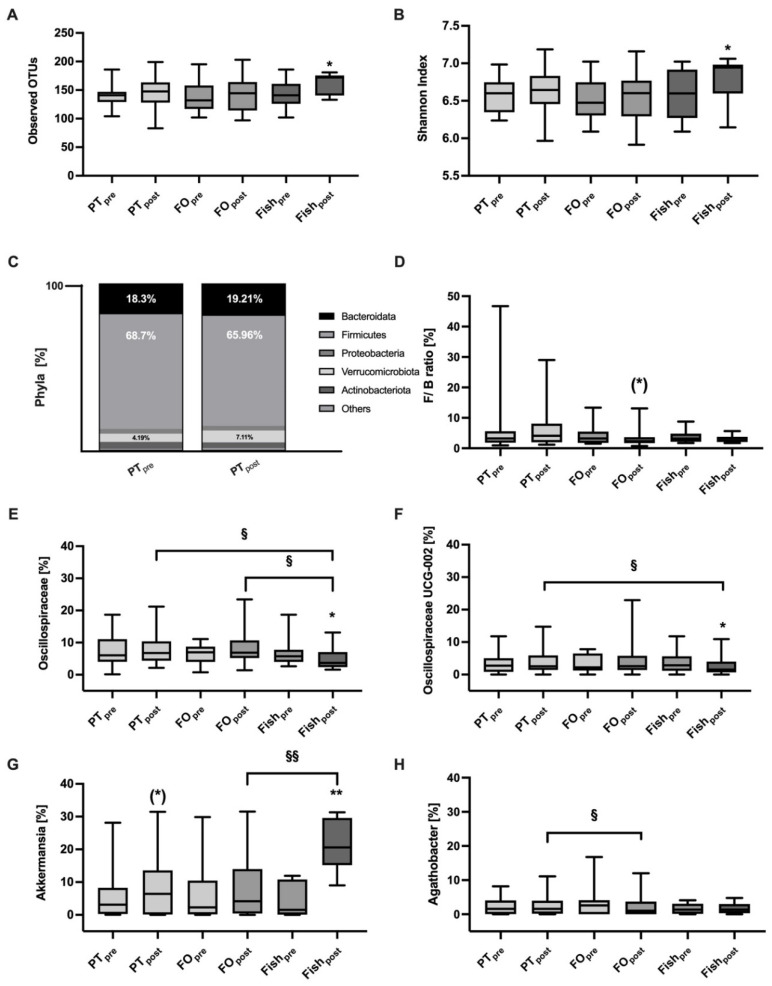
PT did not affect the gut microbiome. The α-diversity (Observed OTU, panel (**A**)) and the Shannon index (panel (**B**)) increase following the fish intervention. Relative bacterial abundancies at the phylum level (panel (**C**)) and the *Firmicutes*/*Bacteroides* (F/B) ratio (panel (**D**)) remained unchanged. Some changes of selected bacterial in family and genus level were found only after fish consumption (panel (**E**–**H**), see Supplementary Table S3). Data are shown as bar plots (panel (**C**)) or as box plots with median, 25% and 75% percentiles, min and max. * indicate differences to “pre”, § indicate differences between different interventions. (*) *p* = 0.1, */§ *p* < 0.05, **/§§ *p* < 0.01.

Analysis of the gut microbiome revealed only minor changes following the consumption of the three study products. The β-diversity (bray-curtis distance) was not affected by the different interventions (data not shown). The α-diversity related to bacterial richness (observed OUT; fish, *p* = 0.02) and the Shannon index (variety; fish, *p* = 0.04) increased only after fish consumption but was not changed by PT or FO exposure (Figure 5A,B). Evenness (equal distribution) was not affected by all three interventions (not shown). We found no changes at the phylum level before and after PT consumption (Figure 5C). Additionally, the ratio of *firmicutes*/*bacteroides* (F/B ratio) was not affected, except for a trend of reduction by FO that could be seen (*p* = 0.1, Figure 5D). At the family level, *Rikenellaceae*, *Christensenellaceae*, *Lachnospiraceae*, *Oscillospiraceae*, *Ruminococcaceae*, *Akkermansiaceae* showed a trend of increasing, except *Lachnospiraceae* which decreased after FO and PT intake, the latter by trend (Supplementary Table S3). Only fish consumption resulted in some changes at the family and genus level. Fish consumption leads to a decrease of the *Oscillospiraceae* family and the UCG-002 genus belonging to *Oscillospiraceae* (Figure 5E,F), and to an increase in the *Lachnospiraceae* family (Supplementary Table S3) and the *Akkermansia* genus (Figure 5G). Additionally, PT induced some increase in *Akkermansia* by trend and *Agathobacter* was higher after PT compared to FO administration (Figure 5H).

## 3. Discussion

The present study is the first in which the unfractionated microalgae PT has been examined in humans. We focused in this pilot trial on acceptance of the product, safety issues, and bioavailability of nutritive components of PT. Our data revealed that the bioavailability of fatty acids such as *n*-3 PUFA and EPA is similar to PT and FO administered in a crossover design, indicating that such fatty acids can be absorbed equally well from milled microalgae biomass suspensions and a commercially available fish oil product. Moreover, we could show that selected carotenoids, such as FX and its metabolites, and β-carotene are bioavailable from PT. The data suggest that PT should be considered for human nutrition in the future. By consuming the entire biomass, which has been only processed by ball milling to disrupt the microalgae cells, the costs of nutrient-rich food made from PT could be kept low. Microalgae such as PT contain for example high amounts of protein and minerals, and we could show in a pre-clinical study that the bioavailability of this protein is high [28]. Based on this data, microalgae such as PT could become a valuable and sustainable new food source, especially for vegetarians and vegans, as well as for people in developing countries.

The present study confirms that PT is an *n*-3 PUFA rich microalgae that contains *n*-3 PUFA at a concentration of 58 mg/g, compared to 21 mg/g in fish. EPA is the main *n*-3 PUFA in PT (53 mg/g), whereas fish contains much less (7 mg/g). For this reason, PT is used at up to 6% in fish meals for feeding Atlantic salmon [33]. Previous studies showed that DHA is well absorbed from oil derived from the microalgae *Schizochytrium* sp. [34], or from *Crypthecodinium cohnii* [35] in a similar range as from classical FO or whole sea fish. Our study extends this finding by showing that EPA and the total *n*-3 PUFA are well absorbed from milled and resuspended PT. The fact that DHA did not increase following PT consumption might be explained due to the minor amounts of DHA in PT compared to FO or fish, and not necessarily because DHA bioavailability is low. However, this must be further evaluated in the future. The observation that EPA increased after fish and FO consumption to a similar degree as after PT consumption, even though fish and FO contained lower amounts of EPA, could be due to retro conversion of DHA to EPA after absorption [36].

Our study provides evidence that PT is a valuable source for EPA, as described before for another diatom, *Odontella aurita*, which has been already authorized as whole algae, although no nutritional human trials are available so far, in which *Odontella aurita* has been tested for safety or bioavailability of its ingredients. The nutritional composition of *Odontella aurita* is quite similar to that of PT [37] and the microalgae have been shown in pre-clinical studies to reduce risk factors for metabolic syndrome [38,39] similar to PT [23]. Although *Odontella aurita* has been approved as whole microalgae, only 0.5–1.5% of it is allowed in food, mainly for flavoring [22]. From such small amounts, no health benefits can be expected. Higher amounts need to be tested in prospective randomized trials.

EPA and DHA are both necessary to increase the total *n*-3 PUFA, therefore PT could be an alternative EPA source and serve together with DHA-rich microalgae like *Schizochytrium* sp, which is mainly used in supplements with *Ulkenia* sp. It has been shown that the regular intake of EPA and DHA leads to a reduction in the *n*-6:*n*-3 ratio of PUFA, which results in higher EPA + DHA concentrations in red blood cell membranes (omega-3 index), considered as beneficial, e.g., to lower inflammatory processes [40]. The Western diet is characterized by a high *n*-6:*n*-3 ratio thought to promote diet-related diseases such as obesity, type 2 diabetes, and cardiovascular disease [41]. The *n*-3 PUFA EPA and DHA not only have anti-inflammatory and anticoagulant effects but also promote the synthesis of specialized pro-resolving mediators crucial for the healing phase and termination of inflammation [42]. A low PUFA *n*-6:*n*-3 ratio is therefore associated with a reduced risk of cardiometabolic diseases [43] and possible prevention for death by COVID-19, as shown recently [44]. Our study shows that the *n*-6:*n*-3 ratio and total *n*-6 PUFA can be reduced not only by the consumption of fish and FO but also by PT consumption.

Another particular feature of diatoms is the high content of FX besides EPA. FX is found in PT, as well as in *Odontella aurita* [37] at concentrations of about 10–20 mg/g dry weight) depending on the culture condition. The potent antioxidant is also found in some brown algae such as *U. pinnatifida* found in Miso soup and Wakame, albeit at lower concentrations (2.7 mg/g) [45]. A good bioavailability is a requirement for beneficial health effects, e.g., anti-cancer and anti-obesity effects [46,47]. Our study demonstrates that FX is absorbed in the intestine and can be detected in plasma; however, only at low levels (Figure 2). A likely explanation for the low plasma concentrations of FX is that it is rapidly metabolized into FXOH and AxA. These two FX metabolites were detected at much higher concentrations compared to FX supporting our hypothesis of a rapid metabolization in the intestine and liver. This hypothesis is also supported by pharmacokinetic studies in rodents [48,49], and a few conflicting studies in humans. One human study reported a maximum concentration (Cmax) of 44 nmol/L FXOH in plasma 4 h after ingestion of 31 mg FX of Kombu extract [50]. Others found only a concentration of 2.7 nmol/L FXOH in normal and overweight humans after ingestion of 2 mg FX from Akamoku oil for eight weeks [51]. A third human trial revealed an increase of 0.8 nmol/L FXOH in plasma after one week of ingestion of 6.1 g FX from Wakame [52].

In our study, ingestion of PT at 30 mg/d FX increased FXOH plasma concentrations to 232 nmol/L after one week and 482 nmol/L after two weeks, suggesting the bioavailability of FX metabolites from PT. Moreover, we detected AxA, another FX metabolite that was not analyzed in the previous human trials, at up to 111 nmol/L. This indicates a further metabolization of FXOH in the liver. Our dosing of FX from PT, which contains about 1% of FX, corresponds to approximately 0.5 mg/kg body weight. In a rodent model, a dose between 1 and 10 mg/kg body weight has been administered for 28 days and no increase in mortality or toxic effects have been observed [53]. FX from macroalgae is approved for supplementation in the EU. For the PT-based oil, which contains about 2% FX, the recommended dose has been set to 437 mg, which corresponds to 10 mg FX because higher doses have not been studied so far. In our study, the higher dose of 30 mg FX and Chlorophyll caused some green discoloration of the feces, but almost no adverse effects and, if at all, only mild abdominal symptoms, such as bloating, stomach pain, belching, constipation, and diarrhea in a few participants. Moreover, we observed no toxic effects in our study, since none of the safety parameters included (γ-GT, AST, ALT, CRP, glucose, uric acid, cholesterol, and triacylglycerols) a change upon intervention. This confirms our previous toxicological studies in mice showing no toxic effects of PT administered at very high concentrations [28].

PT is also a source of lutein/zeaxanthin, which is otherwise found in green vegetables, yellow fruits, and egg yolk [54], and of β-carotene, which is present mainly in carrots. However, according to our data, the bioavailability of lutein/zeaxanthin seems to be low, but this might be a result of the suboptimal timing of our measurements. We measured one and two weeks after the start of the challenge to assess *n*-3 PUFA. For assessment of the lutein/zeaxanthin bioavailability, shorter time intervals might be needed, such as hours or a few days, as performed in classical pharmacokinetic studies. While lutein/zeaxanthin levels hardly changed, we observed an increase in β-carotene levels, but not of retinol, after one and two weeks. The low cleavage of β-carotene to retinol could result from the presence of retinol in the diet [55], but also due to an interaction with other carotenoids such as zeaxanthin [56]. Nevertheless, microalgae are generally good sources of carotenoids. Harvey et al. showed that the cost of producing β-carotene synthetically is 10 times higher than using the microalgae *Dunella salina* [57].

Another ingredient of PT is tocopherol, the amount of which is dependent on the microalgae cultivation conditions [58] and on the processing since it was added as a fat-soluble antioxidant during the cell disruption. The microalgae PT contains all tocopherols and γ-tocotrienol. During the study, the subjects consumed ~2.5 mg of total vitamin E daily just by PT intake, which covers 16.5% of the daily requirement. No increase of α- and γ-tocopherol levels were measured after two weeks of consumption of PT, which does not exclude some tocopherol uptake at earlier time points. More detailed pharmacokinetic studies are required to assess bioavailability here since vitamin E concentrations are regulated by the hepatic α-tocopherol transfer protein and undergo metabolic degradation to short-chain metabolites within due time.

Gut-related parameters were carefully assessed in our study for two reasons. First, microalgae components such as fibers might induce some adverse effects, such as bloating, but also increase the production of SCFA. Secondly, gut barrier function or commensal microbiota could be affected. As reported before, PT induced some mild GI symptoms at slightly higher rates compared to FO or fish, but no effects on SCFA concentrations in feces. It is possible that the number of fibers (15 g fibers per 100 g PT, corresponding to <1 g fibers per day) and *n*-3 PUFA may not be enough to alter SCFA production by the commensal microbiota. Detailed microbiome analysis at the 16S RNA level revealed only minor changes that were statistically significant only after fish consumption for a few selected bacterial genera such as *Akkermansia*, which increased after fish and by trend after PT consumption, but not after FO consumption. Such results might become different if patients, e.g., with inflammatory bowel diseases [59], instead of healthy subjects are studied.

## 4. Materials and Methods

### 4.1. Participant Selection

Twenty-five healthy adults (15 females and 7 males, age 18–50 years) were recruited for the study (Figure 1) and provided written informed consent. Exclusion criteria were pregnancy, breastfeeding, use of medications, such as antibiotics, intestinal therapeutics, and immunosuppressants. Relevant violations of the dietary protocol did not occur. The study was conducted at the University of Hohenheim in 2020 according to the Declaration of Helsinki, has been approved by the local Ethical Committee (Ethik-Kommission der Landesärztekammer Baden-Württemberg), and was registered at ClinicalTrials.gov (NCT04288544). The study was planned as a proof-of-principle pilot study; therefore, no formal calculation of case numbers was performed. According to other studies in this area, our goal was to have at least 20 individuals available for analysis.

### 4.2. Study Design and Intervention

The study was designed as a randomized, single-blind, monocentric intervention trial in a crossover design (Figure 1) with 11 study visits. Participants were randomly assigned to group 1, starting with the microalgae PT (5.3 g per day) administered for two weeks (PTpost) and a FO capsule intake (one per day) for two subsequent weeks (FOpost), or to group 2 receiving the same intervention in the opposite order (FOpost and PTpost), with a two-week washout period before the first (pre-intervention 1) and the second phase (pre-intervention 2) of intervention. Due to restrictions related to the COVID-19 pandemic, all subjects underwent a five-week break after the first intervention phase (post-intervention 1) followed by the second washout phase (pre-intervention 2) before the second intervention.

All subjects who passed the crossover study with two interventions PT and FO were invited for a third intervention phase of two weeks without PT or FO, but fish as a positive control. These individuals (nine out of 22) were served four servings of salmon (125 g and 60 g in the first week, 60 g and 125 g in the second week) resulting in 185 g salmon per week (post-intervention fish; Fishpost). These chosen fish doses per week resulted in an EPA + DHA challenge similar to that achieved by PT or FO intervention in the previous study parts.

Fasting blood samples were collected at all-time points: at the baseline, after a two-week washout period (pre-intervention 1, 2, and 3), after one week of the taking study product, and two weeks of the taking study products (post-intervention 1, 2 and fish) at all three phases. Fecal samples were collected at pre-intervention 1, 2 (PTpre, FOpre) and post-intervention 1, 2 (PTpost, FOpost) and fish (Fishpost). During the whole study period, participants were asked to follow their habitual diet with some restrictions. They had to avoid foods containing *n*-3 PUFA, such as fish, vegetable oils, vegetables, seeds, and nuts as well as *n*-3 PUFA-fortified foods. All participants underwent study-specific diet counseling and were instructed to take the study food in addition to their habitual diet.

### 4.3. Subject Characterization

Twenty-two out of 25 participants (10 men and 15 women) completed the study, namely the two major intervention phases PT and FO. Only three volunteers (three men) dropped out after four weeks for reasons not related to the study (COVID-19 pandemic and personal reasons). All 22 participants were invited to participate in a study extension with fish exposure instead of PT or FO. Nine of them agreed and completed the fish phase immediately after the previous study phase with a two-week washout in-between. Baseline characteristics of study participants are shown in Table 3. None of the participants showed any major changes in their health status during the study. Group 1 started with PT intake, group 2 started with FO intake and at the baseline, there was no difference between the study groups 1 and 2 and no changes were seen during the intervention (Table 3).

The compliance was checked every two weeks and revealed that all participants consumed the intervention products according to the protocol. All participants followed the instructions not to eat any *n*-3-rich foods during the washout and interventional phases. To record food intake in detail, a Food Frequency Questionnaire (FFQ) was requested during the whole study time, there was no difference between the study groups 1 and 2 (Table 3).

### 4.4. Study Products

PT SAG 1090-1b was cultivated in flat-panel airlift photobioreactors at Fraunhofer CBP as described previously [29]. After harvesting, the biomass was concentrated to 250 g/L using a centrifuge (Clara 20, Alfa Laval, Glinde, Germany) and stored at −20 °C until further processing. After thawing, the biomass was diluted with deionized water to 100 g/L and subsequently, the cells were disrupted in a ball mill according to Derwenskus et al. [60]. To minimize oxidation during cell disruption, 0.2 g/L of all rac-α-tocopherol was added (Roth Inc., Karlsruhe, Germany). The biomass was freeze-dried for 36 h at −20 °C and 0.1 mbar (VaCo 5. Zirbus, Tiel, The Netherlands) and stored at −20 °C protected from light until further use.

The amount of PT and FO was chosen based on the national *n*-3 PUFA/EPA + DHA recommendation of 250 to 300 mg per day [17]. Accordingly, the subjects received 5.3 g PT daily (Table 4) divided into two servings, one at noon and one in the evening, delivering a total of 305 mg of *n*-3 PUFA. For comparison, we administered FO capsules resulting in an intake of a similar amount per day. Subjects took one commercially available capsule daily in the evening (softgel capsule “Essential Omega-3” containing 310 mg *n*-3 PUFA and antioxidants; Myprotein, Manchester, UK).

For the third intervention, we selected salmon (Salmo salar) from Norway from aquaculture, because this oily fish is rich in *n*-3 PUFA and a natural source for EPA and DHA. Since EPA and DHA are the essential *n*-3 PUFA, we adapted the number of salmon administered in the study to an EPA and DHA intake of about 300 mg per day. Since fish, in contrast to PT and FO, contains another *n*-3 PUFA in addition to EPA and DHA, the total *n*-3 PUFA intake during the fish intervention was higher than during the PT or FO intervention (Table 4). This approach resulted in the consumption of 185 g salmon per week, divided into 2 servings (125 g and 60 g). The frozen salmon of organic quality was purchased from a supermarket (Aldi Süd, Harsum, Germany). The detailed composition of the study products is shown in Supplementary Table S1.

### 4.5. Blood Plasma, Serum Measurements, and Fecal Samples

Blood samples were collected in two ethylenediaminetetraacetic acid (EDTA)-coated tubes and one serum tube. One plasma tube was used for blood count analysis (Sindelfingen laboratory GbR, Sindelfingen, Germany). The other one was centrifuged at 500 g for 7.5 min at 15 °C followed by separation of plasma, which was stored at −80 °C until analysis for fatty acids, carotenoids, retinol, tocopherols, and lipopolysaccharide-binding protein (LBP) quantification.

Serum tubes were centrifuged 15 min at 3000× *g*, and serum was used for quantification of γ-gamma-glutamyl transferase (γ-GT), aspartate aminotransferase (AST), alanine transaminase (ALT), c-reactive protein (CRP), plasma glucose, uric acid, triacylglycerols (TAG), cholesterol (Chol), High-density lipoprotein (HDL) and Low-density lipoprotein (LDL) (Sindelfingen laboratory GbR).

Stool samples were collected prior to the study date (max. 2 days before) in two tubes and stored at −20 °C at home or transported directly to the laboratory. In our laboratory, the two tubes were stored at −80 °C until further analysis. One tube was used to measure the gut barrier marker zonulin and SCFA, the other tube was used for DNA isolation for gut microbiome sequencing.

### 4.6. Quantification of Plasma Fatty Acids

In brief, 2 µL 10,11-dichloro-undecanoic acid (11:0) as internal standard and 2 mL methanol (Carl Roth GmbH, Karlsruhe, Germany) with 1% sulphuric acid for transesterification according to the method of Thurnhofer and Vetter was added to 100 µL plasma as described [61]. Next, 5 μL tetrade-canoic acid-EE (14:0-ethyl ester) was added before the samples were analyzed by gas chromatography with mass spectrometry on a 5890 series II/5972A system (Hewlett-Packard, Waldbronn, Germany) operated in selected ion monitoring mode according to Thurnhofer et al. [32,62].

### 4.7. Quantification of the Carotenoid FX and Its Metabolites FXOH and AxA in Plasma

To measure FX and their metabolites FXOH and AxA, 100 µL of human plasma was mixed with 200 µL of ethanol/butanol (50/50 (*v*/*v*)) containing 5 mg butylated hydroxytoluene. After vigorous mixing and centrifugation (17,000× *g* and 4 °C, 10 min) (Heraeus Fresco 17, Thermo Fischer Scientific, Waltham, MA, USA), 10 μL of clear supernatants were injected into a Shimadzu HPLC system (Mc Kinley Scientific, New York, NY, USA) with a F5 reversed-phase column (2.6 µm F5 100 Å 150 × 4.6 mm, Kinetex, Phenomenex Ltd., Aschaffenburg, Germany) maintained at 40 °C and an ultraviolet detector set to 450 nm. A mixture of methanol/water (85/15 (*v*/*v*)) with a flow rate of 1.0 mL/min for 15 min was used as mobile phase. The autosampler was kept at 15 °C and quantification was achieved using authentic commercial standards (FX purity ≥ 95%; FXO and AxA purity ≥ 97%, Merck Group, Darmstadt, Germany) diluted in ethanol. The method was tested for linearity, sensitivity, and selectivity before sample analysis.

### 4.8. Quantification of the Carotenoids Lutein/Zeaxanthin, Lycopenes, β-Cryptoxantins and α/β-Carotene, Retinol, and α/γ-Tocopherol in Plasma

The extraction of other carotenoids, retinol, and tocopherols from human plasma was performed as previously described [63] with some modifications. Forty microliters of plasma were mixed with 200 µL of ethanol/butanol (50/50 (*v*/*v*)) containing 12 µL beta-apo-8′-carotenal-methyloxime/100 mL (internal standard). After vigorous mixing and centrifugation (17,000× *g* and 4 °C. 10 min) (Thermo Fischer Scientific), 20 μL of clear supernatants were injected into a Shimadzu HPLC system (see above) with a ReproSil 80 ODS-2 column (3 µm, 250 × 4.6 mm) (Dr. A. Maisch GmbH, Ammerbuch-Entringen, Germany) maintained at 40 °C. A mixture of acetonitrile/1,4-dioxane/methanol (82/15/3; *v*/*v*) containing 100 mmol/L ammonium acetate and 0.1% trimethylamine, at a flow rate of 1.5 mL/min for 20 min was used as mobile phase and autosampler temperature was maintained at 5 °C. Detection of carotenoids was performed using an ultraviolet detector set to 450 nm while quantification of retinol and tocopherols used a fluorescence detector (Ex/Em at 325/470 nm for retinol for 0–5 min and Ex/Em at 296/325 nm for α/γ-tocopherol for 5–20 min). Quantification of all analytes was performed using authentic commercial standards corrected by the internal standard.

### 4.9. Quantification of Tocopherol, Tocotrienol, and Carotenoids in PT and FO by HPLC

In PT and FO, Vitamin E was determined by hexane-based extraction. PT determination was performed from freeze-dried material. For FO preparation ten capsules were chosen and mixed homogenously. Later triplicate samples of 100 mg PT or 25 mg FO were saponified with KOH, neutralized, extracted with hexane, and later vitamin E was resuspended in ethanol as previously described [64]. For measurement of vitamin E, 20 µL of the ethanolic suspension were injected into a Jasco HPLC system (JASCO Deutschland GmbH, Pfungstadt, Germany) with PFP reverse phase column (2.6 µm PFP 100 Å 100 × 4.6 mm, Kinetex, Phenomenex Ltd., Aschaffenburg, Germany) maintained at 40 °C using a mobile phase methanol/water (85:15 (*v*/*v*)) with a flow rate of 1.2 mL/min for 30 min. Autosampler temperature was set to 5 °C and quantification was performed using a fluorescence detector with excitation/emission wavelengths of 296/325 nm against authentic standards for of RRR-α-tocopherol, RRR-β-tocopherol, RRR-δ-tocopherol, RRR-γ-tocopherol (purity ≥ 95 %, Merck Group), α-tocotrienol, β-tocotrienol, δ-tocotrienol, γ-tocotrienol (purity ≥ 97 %, Merck Group) diluted in ethanol. The method of quantification of FX and β-carotene in PT was previously described by Derwenskus et al. [60].

### 4.10. SCFA Analysis from Stool Samples

Raw fecal samples were homogenized, weighed (ca. 400 mg), diluted 1:4 with distilled water and 100 µL 50% phosphoric acid (Carl Roth GmbH, Karlsruhe, Germany) were added. Samples were homogenized with a whirlmix and centrifuged (20,000× *g* at 4 °C, 20 min) twice (5417R, Eppendorf, Hamburg, Germany). The supernatant was drawn up and filtered with a syringe filter with glas fiber (WIC 79545, Wicom, Heppenheim, Germany) to an autosampler glas (WIC 42100 with crimp caps, Wicom) with Micro Inserts (No 548-00060, VWR International GmbH, Darmstadt, Germany). With a capillary gas chromatograph (Clarus 690, Perkin-Elmer, Waltham, MA, USA), using a liquid autosampler with a capillary column (Cat. # N9316354, Perkin Elmer) with standards (Volatile Free fatty acid Mix CRM46975, Merck Schuchhardt OHG, Hohenbrunn, Germany), 1 µL filtrate was analyzed. For data integration, the software total-Chrome Version 6.3.4 (Perkin-Elmer, Waltham, MA, USA) was used. For fecal dry mass quantification, 200 mg fecal samples were weighed and dried for 12 h at 103 °C. SCFA data are expressed in relation to dry mass and identified by comparing the retention times of the respective peaks in the sample and standard chromatograms.

### 4.11. Analysis of Intestinal Permeability Markers Plasma Lipopolysaccharide-Binding Protein (LBP) and Fecal Zonulin

Both zonulin and LBP were measured using commercial enzyme-linked immunosorbent assay kits (K5600, KR6813, Immundiagnostik AG, Bensheim, Germany) following the manufacturer’s protocols. The fecal samples were diluted to the working concentration in sample buffer using stool sample tubes (K6998SAS; Immundiagnostik AG, Bensheim, Germany) and for LBP analysis, 10 µL of blood plasma was used and processed as described [32].

### 4.12. Gut Microbiome Analysis

Bacterial DNA was extracted from fecal samples using the QIAamp Fast DNA Stool Mini Kit (Cat. No. 51604, Germantown, MD, USA) following the manufacturer’s protocol. 16S Ribosomal RNA (rRNA) gene amplicons of 2 × 300 bp length were sequenced on the MiSeq platform at the University of Minnesota Genomics Center, targeting the V5–V6 regions using the primers V5F and V6R. For evaluation, the DADA2 and QIIME2 pipelines were used. First, paired-end reads were merged, demultiplexed, and quality control was conducted (length = 250 bp, mean sequence quality score ≥ 30). We refer to these adapted paired-end reads as amplicon sequences variants (ASVs). Samples with sequencing read below 8080 were excluded, resulting in 18 individuals within PT and FO and 9 individuals within fish intervention which were included in the microbiome analyses. After that, the data was converted into relative abundance and below 0.15% in all samples were removed. This resulted in 102 ASVs. Microbial alpha and beta-diversity were measured and taxonomic categories from the phylum to species (ASV) levels were categorized using a pre-trained Naive Bayes classifier 2 based on Silva 138 99% OTUs database [65].

### 4.13. Statistical Analyses

First, all parameters were tested for normal distribution using the Kolmogorov-Smirnov test. For parametric parameters a one-way ANOVA was conducted with the Geisser-Greenhouse correction and for multiple comparisons between visits (baseline, pre- and post-intervention) within one intervention (PT, FO, fish) the Tukey test was used. Non-parametric parameters were tested with the Friedman test and, as a post hoc test, Dunn’s multiple comparison test. To compare different interventions at given time points the two-tailed paired *t*-test was done for parametric variables and the paired Wilcoxon-matched-pairs-rank-test was used for non-parametric variables. Gut barrier marker, SCFA, and gut microbiome were measured at two time points and were also analyzed by the two-tailed paired t-test or the paired Wilcoxon-rank-test. For Fish pre phase, no stool samples were given, Fishpre represents the value at pre-intervention 2 before the fish phase- either FOpre (*n* = 5) or PTpre (*n* = 4) of the 9 participants. All data were analyzed per protocol with the exclusion of drop-outs after interventions 1 and 2. All statistical analyses were performed using GraphPad Prism version 9.0.1.

## 5. Conclusions

In conclusion, the study shows that PT is safe to consume for humans. EPA and FX are accessible from PT without processing the biomass, other than ball milling. In particular, the *n*-3 PUFA, and especially EPA, were absorbed in similar levels as those obtained from FO and fish. The carotenoids β-carotene and FX were also well absorbed, and the metabolites of FX were measured at higher concentrations in plasma than FX itself. This suggests that PT could serve as a novel food source in the future. Other potentially valuable components, such as proteins, need to be investigated in future studies. The taste and texture of the microalgae product need to be further improved for bringing this sustainable food source to the market.

## Figures and Tables

**Table 1:** Adverse effects during the intervention.

Side Effects	PT (*n* = 22) Diary|Protocol			FO (*n* = 22) Diary|Protocol			Fish (*n* = 9) Diary|Protocol		
	Minimal	Mild	Severe	Minimal	Mild	Severe	Minimal	Mild	Severe
Bowel problems	2|6	-	-	1|6	-	-	-	-	-
Bloating	1|6	.	-		1|1	-	-	-	-
Stomach pain	2|2	-	-	-	-	-	-	-	-
Constipation	1|2	-	-	-	-	-	-	-	-
Stool discoloration	0|2	-	-	-	-	-	-	-	-
Increased bowel movements	1|2	-	-	-	1|2	-	-	-	-
Belching (at least 1×)	1|2	-	-	-	7|7	-	-	-	-
Headache	0|2	-	-	1|0	-	-	-	-	-
Increased skin impurities	0|1	-	-	-	-	-	-	-	-
Increased feeling of thirst	0|1	-	-	-	-	-	-	-	-
Reduced appetite	0|1	-	-	-	-	-	-	-	-

**Table 2:** Effect of interventions on laboratory parameters.

Blood Count	Baseline (V1)	PT_pre_ (V2/V6)	PT_post_ (V4/V8)	FO_pre_ (V2/V6)	FO_post_ (V4/V8)	Fish_pre_ (V9)	Fish_post_ (V11)
γ-GT [mg/L]	14.4 ± 4.9	14.1 ± 4.4	13.6 ± 4.0	14.0 ± 4.4	14.4 ± 5.2	11.4 ± 3.2	12.4 ± 2.7 *
AST [mg/L]	21.7 ± 5.0	21.5 ± 3.9	25.0 ± 12.5	21.6 ± 4.8	24.0 ± 10.3	23.6 ± 4.2	23.3 ± 7.8
ALT [mg/L]	19.3 ± 12.8	16.8 ± 5.5	17.0 ± 7.8	15.5 ± 5.8	21.1 ± 21	14.8 ± 5.9	12.9 ± 3.3 ^#^
CRP [mg/L]	1.1 ± 1.4	1.0 ± 0.9	1.1 ± 1.0	0.8 ± 0.8	0.8 ± 0.8	0.8 ± 0.7	1.1 ± 1.0
Glucose [mg/dL]	81.5 ± 8.9	83.0 ± 7.0	81.1 ± 6.0	83.8 ± 6.0	81.1 ± 5.7	77.8 ± 3.7	81.9 ± 3.1
Uric acid [mg/dL]	4.5 ± 0.9	4.4 ± 0.8	4.8 ± 1.1 **	4.6 ± 1.1	4.7 ± 1.1	4.4 ± 0.5	4.3 ± 0.6
Chol [mg/dL]	187 ± 37	186 ± 40	195 ± 31	188 ± 37	180 ± 30 ^§§^	191 ± 38	192 ± 36
TAG [mg/dL]	78.2 ± 23.3	91.2 ± 49.5	94.8 ± 43.7	84.2 ± 27.5	75.0 ± 28.6 ^§§^	91.3 ± 69.9	78.3 ± 36.7
HDL Chol [mg/dL]	58.7 ± 12.3	59.3 ± 11.1	62.3 ± 12.0 ^#^	61.5 ± 13.7	58.9 ± 11.9 ^§§^	63.9 ± 10.6	66.3 ± 8.7
LDL Chol [mg/dL]	115 ± 26.2	106 ± 27.2	115 ± 22.7	110 ± 26.6	104 ± 21.3 ^#§§^	116 ± 26.4	115 ± 24.0
LDL/HDL- ratio	2.1 ± 0.7	1.9 ± 0.6 ^#^	2.0 ± 0.6	1.9 ± 0.6 ^##^	1.9 ± 0.5 ^##§^	1.9 ± 0.3	1.8 ± 0.3 *
Chol/HDL- ratio	3.3 ± 0.8	3.3 ± 0.8	3.2 ± 0.8	3.2 ± 0.7	3.2 ± 0.7	3.1 ± 0.4	2.9 ± 0.4 **

Values are expressed as arithmetic mean ± SD from 22 (PT, FO), or 9 (Fish intervention) participants. Analyses were measured at different time points determined before (“pre”) and after (“post”) intervention. Abbreviations: PT, Interventions with *Phaeodactylum tricornutum*; FO, Intervention with fish oil; Fish, Intervention with salmon; γ-GT, gamma-glutamyl transferase; AST, aspartate aminotransferase; ALT, alanine transaminase; CRP, c-reactive protein; Chol, cholesterol; TAG, Triacylglycerols; HDL, High-density lipoprotein; LDL, Low-density lipoprotein. Statistics: * indicate differences to “pre”, ^#^ indicate differences to baseline, ^§^ indicate differences between PTpost and FOpost. */^#^/^§^ *p* < 0.05, **/^##^/^§§^ *p* < 0.01.

**Table 3:** Baseline characteristics of the study participants.

Parameter	All (*n* = 22)	Group 1 (*n* = 11)	Group 2 (*n* = 11)
Age [years]	25.7 ± 5.7	26.5 ± 7	24.8 ± 3.3
Female/male [n]	15|7	10|3	5|4
Body Weight [Kg]	65.9 ± 10.7	65.8 ± 12.8	68.2 ± 9
Height [m]	1.74 ± 1	1.72 ± 0.09	1.78 ± 0.09
BMI [kg/m^2^]	21.5 ± 2.0	22.0 ± 2.6	21.5 ± 1.9
Vegetarian|Vegan|	2|2|	2|0	0|2
Hemoglobin [g/dL]	14.4 ± 1.3	14.3 ± 1.1	14.4 ± 1.6
Hematocrit [%]	42.5 ± 2.9	42.2 ± 2.2	42.5 ± 3.8
Erythrocytes [cells/pL]	4.9 ± 0.3	4.9 ± 0.2	4.9 ± 0.4
Leucocyte [cells/nL]	5.6 ± 0.4	6.2 ± 1.2	5.3 ± 2.3
Platelet count [cells/nL]	240.8 ± 59.6	250.1 ± 54.5	245.5 ± 68
TSH [mU/L]	1.6 ± 0.6	1.8 ± 0.7	1.5 ± 0.4
Insulin [µE/mL]	7.1 ± 3.3	8.4 ± 3.6	6.0 ± 2.9
HOMA-IR	1.4 ± 0.7	1.7 ± 0.8	1.2 ± 0.7
Dietary intake (FFQ) [g/day]			
Fat	49.2 ± 23.7	44.8 ± 18.9	55.5 ± 29.4
Saturated fatty acids	22.23 ± 11.0	19.7 ± 7.9	25.9 ± 14.11
Unsaturated fatty acids	7.6 ± 4.1	7.0 ± 3.4	8.5 ± 5.0
Short chain fatty acids	1.2 ± 0.8	0.9 ± 0.4	1.4 ± 1.1
Long chain fatty acids	43.40 ± 20.9	39.6 ± 17.13	48.9 ± 25.5
Cholesterol	191.8 ± 109.2	177.2 ± 104.4	212.9 ± 118.7

Values are expressed as Mean ± standard deviation (SD) which is expressed in absolute numbers. Abbreviations: PT, *Phaeodactylum tricornutum*; FO, fish oil; BMI, body mass index; HOMA-IR, Homeostasis Model Assessment for Insulin Resistance; FFQ, Food Frequency Questionnaire. Group 1 started with PT intake; group 2 started with FO intake. Statistics: Comparison of group 1 and 2 revealed no difference for all parameters listed in the table (*p* > 0.05, paired *t*-test).

**Table 4:** Daily intake of omega-3 and omega-6 fatty acids by the study participants.

Fatty Acids [mg/day]	PT	FO	Fish
*n*-3 PUFA	305	310	554 *
EPA + DHA	286	300	299 *
20: 5 *n*-3 (EPA)	282	178	198 *
22:6 *n*-3 (DHA)	5	122	101 *
18:3 *n*-3 (ALA)	0.9	1.8	94.1 *
18:4 *n*-3	0.7	0.2	58.9 *
22:5 *n*-3	9.5	7	100 *
16:2 *n*-6	13.4	2.8	0.6 *
18:2 *n*-6	3.8	5.3	34.9 *
18:3 *n*-6	1.2	0.9	0.1 *
20:3 *n*-6	0.1	0.3	0.2 *
20:4 *n*-6	3.7	0	50.2 *

Abbreviations: PT, Intervention with the microalgae *Phaeodactylum tricornutum*; FO, Intervention with fish oil; Fish, Intervention with salmon served as two servings per week; *n*-3 PUFA, total amount of polyunsaturated omega-3 fatty acids; EPA, Eicosapentaenoic acid; DHA, Docosahexaenoic acid, ALA, alpha-linolenic acid * Average daily intake calculated based on weekly amount.

## Data Availability

Data are available upon justified request to the corresponding author.

## Supplementary Materials

The following are available online at https://www.mdpi.com/article/10.3390/md19120700/s1, Table S1: Nutrient composition of PT, FO and fish (salmon) used in the study; Table S2: Plasma fatty acids concentrations during each of the experimental phases (in percent %); Table S3: Bacterial taxa in feces at pre- and post-intervention.
