# Supplementary material for: Oral Bioavailability of Omega-3 Fatty Acids and Carotenoids from the Microalgae Phaeodactylum tricornutum in Healthy Young Adults
Source: Mar Drugs. 2021 Dec 10;19(12):700. doi: 10.3390/md19120700 (PMC8709223; doi:10.3390/md19120700)
Supplement: Supplementary file 1 [file marinedrugs-19-00700-s001.zip › marinedrugs-1491259-supplementary.pdf]

Supplementary Materials

Table S1. Nutrient composition of PT, FO and fish (salmon) used in the study

Nutrient composition	PT	FO	Fish (salmon)
[KJ/100g]	1691#		741
[kcal/100g]	405#		178
Protein [g/100g]	47.2#	-	18
Carbohydrate [g/100g]	9.00#	-	<0.5
Sugars [g/100g]	0.78#	-	<0.5
Dietary fibre [g/100g]	15	*	*
Fat. Total [g/100g]	16.6#	5.2	13.6
n-3 PUFA [mg/g]	57.5± 2.7	310.4± 3.4	21
n-6 PUFA [mg/g]	4.2± 0.03	9.3± 0.5	4.1
PUFA n-6: n-3 ratio	0.07± 0	0.03± 0	0.2
EPA [mg/g]	53.2± 2.9	178.0± 2.0	7.5
DHA [mg/g]	0.89± 0.07	122.5± 2.5	3.8
EPA+ DHA [mg/g]	54.0± 2.8	300.5± 4.5	11.3
Vitamin E. [mg/ kg]	466± 30,9	328.3± 4.8	*
α-T. [mg/ kg]	454± 30,1	309.4± 4.1	*
β-T. [mg/ kg]	9.4± 0,6	nd	*
γ-T. [mg/ kg]	1.4± 0,4	17.5± 4.7	*
δ-T. [mg/ kg]	0.2± 0,0	1.2± 0.5	*
γ-Tocotrienol [mg/ kg]	1.0 ± 0,1	nd	*
**Carotenoids**			
Fucoxanthin [mg/g]	9.2	*	*
β-carotene [mg/g]	1.84	*	*

Values are expressed as mean with SD for the microalgae *Phaeodactylum tricornutum* (PT), fish oil (FO) and fish. PT and FO was measured by our lab and fatty acids of fish, #measured by GBA group for bioanalytic (Hamburg, Germany) and fish amounts (without fatty acids) are from food composition and nutrition tables (Souci, Leipnitz-Gemeinschaft, Freising, Germany). Vitamin E means a sum of all Tocopherols and γ-Tocotrienol measured and shown. Abbreviations: n-3 PUFA, omega-3 polyunsaturated fatty acids; EPA, Eicosapentaenoic acid; DHA, Docosahexaenoic acid; T., Tocopherol. * no data measured.

Table S2. Plasma fatty acids concentrations during each of the experimental phases (in percent %)

Fatty acids (%)	Baseline (V1)	PTpre (V2/ V6)	PTpost (V4/ V8)	FOpre (V2/ V6)	FOpost (V4/ V8)	Fishpre (V9)	Fishpost (V11)
SFA	26.4± 2.7	27.1± 3.0	26.7± 2.8	26.5± 2.6	26.7± 2.6	28.5± 3.6	28.28± 3.33#
UFA	73.6± 2.7	72.9± 3.0	73.3± 2.8	73.5± 2.6	73.3± 2.6	71.5± 3.6	71.7± 3.3#
MUFA	23.6± 4.7	24.0± 4.3	23.7± 4.4	23.9± 4.9	23.5± 5.0	22.2± 3.3	21.0± 1.3
PUFA	50.1± 4.1	48.9± 4.8	49.6± 4.1	49.6± 4.4	49.8± 4.6	49.2± 6.7	50.7±4.4
PUFA n-3	8.8±4.2	8.0± 4.1##	9.3± 3.3**	8.1± 4.2#	9.4± 3.4***	8.8± 5.4#	11.3± 4.6**§
PUFA n-6	42.4± 4.6	41.9± 5.2	40.9±4 .2	42.5± 4.70	41.0± 4.5	42.6± 6.0	41.4±3.9
n-6: n-3	5.5± 2.1	5.9± 2.1	4.7± 1.5**	6.0± 2.64	4.65± 1.5##**	5.5± 2.0	3.9±1.3##**
18:2 n-6	31.4± 5.4	30.4± 5.6	30.0± 4.8	30.7± 4.6	29.8± 4.8	31.2± 4.8	29.9± 3.2
18:3 n-3	0.74± 0.4	0.49±0.2#	0.47± 0.2##	0.46± 0.2##	0.48± 0.2##	0.48± 0.2	0.47± 0.2
20:4 n-6	8.55± 1.5	8.82± 1.6	9.0± 1.6	9.01± 1.3	9.1± 1.4	8.62± 1.6	8.60± 1.1
20:5 n-3 (EPA)	0.54± 0.3	0.4± 0.2#	1.3± 0.4###***	0.4± 0.2	1.0± 0.3###***§§§	0.4± 0.2	1.4± 0.5###***§§§
22:6 n-3 (DHA)	6.01± 1.9	5.60± 1.5	5.42± 1.6	5.69± 1.6	6.40± 1.4**§§	5.29± 1.4	7.0± 1.3#***§§§
EPA+DHA	6.5± 2.1	6.0± 1.6	6.7± 1.6	6.1± 1.6#	7.3± 1.6***	5.7± 1.4	8.3± 1.6###***§§

Values are expressed as Mean % ± SD from 22 (PT, FO), or 9 (Fish intervention) participants. Analyses were measured at different time points as indicated. Abbreviations: PT, Interventions with *Phaeodactylum tricornutum*; FO, Intervention with fish oil; Fish, Intervention with salmon; SFA, short chain fatty acids; UFA, unsaturated fatty acids; MUFA, Monounsaturated fatty acids; PUFA, Polyunsaturated fatty acids; n-3 PUFA, omega-3 polyunsaturated fatty acids; EPA, Eicosapentaenoic acid; DHA, Docosahexaenoic acid. Statistics: * indicate differences to "pre", # indicate differences to baseline, § indicate differences to PTpost. #/§ $p < 0.05$, **/##/§§ $p < 0.01$, ***/###/§§§ $p < 0.001$.

Table S3. Bacterial taxa in feces at pre- and post-intervention

Treatment	PTpre (V2/ V6)	PTpost (V4/ V8)	FOpre (V2/ V6)	FOpost (V4/ V8)	Fishpre (V9)	Fishpost (V11)
**Phylum**						
Bacteroidetes (B)	19.9± 11.2	18.7± 11.9	20.4± 10	24.6± 11.7	21.7± 8.1	23.6± 5.9
Firmicutes (F)	66.6± 12.9	65.8± 15.1	65.0± 13.8	59.4± 11.3	66.1± 9.5	65.1± 8.6
F/B ratio	6.2± 10.4	6.1± 6.8	4.3± 3.4	3.3± 2.9(*)	3.7± 2.2	3.03± 1.2
Verrucomicrobiota	5.5± 6.3	7.9± 9.2	7.0± 10.1	7.2± 8.7	6.4± 8.8	4.3± 5.1
**Class**						
Bacilli	9.0± 8.8	8.1± 6.4	7.6± 5.4	5.5± 3.3	8.5± 5.3	8.4± 4.7
Clostridia	57.5± 11.0	54.8± 13.6	55.9± 12.2	52.2± 10	55.1± 10.5	55.2± 9.8
Verrucomicrobiae	5.4± 6.5	8.3± 9.4	6.9± 10.1	7.2± 8.7	6.4± 8.8	4.2± 5.0
**Order**						
Lachnospirales	23.0± 9.2	20.7± 6.8	21.9± 8.4	18.6± 7.6§	16.4± 6.8	21.0± 4.3*
Oscillospirales	24.8± 6.3	25.2± 7.9	26.1± 8.3	24.8± 6.7	29.3± 9.4	24.7± 7.7*
**Family**						
Rikenellaceae	3.8± 3.3	4.8± 3.8	4.5± 3.3	5.1± 2.7	5.5± 2.6	5.3± 1.3
RF39_*Bacilli*	2.7± 4.0	4.6± 6.3	3.2± 5.0	2.1± 2.5	5.1± 5.6	3.8± 3.7
Christensenellaceae	2.6± 2.0	3.4± 3.9	2.9± 3.2	2.9±3.1	2.4± 1.9	4.2± 4.3
Lachnospiraceae	23.2± 9.3	20.8± 6.8	22.1± 8.5	17.9± 8.5*§	16.5± 6.8	21.1± 4.3*
Oscillospiraceae	7.3± 5.0	7.7± 4.7	6.4± 3.0	8.2± 5.6	6.9± 4.8	4.9± 3.8*§
Ruminococcaceae	12.6± 6.2	13.9± 7.5	15.0± 8.3	11.8± 4.8	17.8± 9.5	15.9± 8.6
Akkermansiaceae	5.1± 6.5	7.7± 9.1	6.9± 10.1	7.4± 8.7	6.5± 8.8	4.2± 5.0
**Genus**						
Bacteroides	7.7± 6.0	8.6± 6.5	8.1± 5.8	12.0± 8.1	10.5± 4.6	11.4± 4.7
Agathobacter	2.3± 2.5	2.7± 3.3	3.0± 4.0	2.2± 2.9§	1.6± 1.5	1.6± 1.6
Alistipes	3.8± 3.5	4.8± 4.0	4.9± 3.7	5.3± 3.2	5.2± 3.1	5.1± 2.0
Bacilli RF39	2.9± 4.3	4.9±6.6	3.4± 5.2	2.2± 2.7	5.3± 5.8	4.1± 3.9
Roseburia	2.7± 2.7	3.0± 1.9	4.1± 3.6	3.1± 3.2	4.7± 5.1	4.8± 4.4
*Oscillospiraceae_* UCG-002	3.4± 3.2	3.9± 3.8	3.3± 2.7	3.9± 4.8	3.6± 3.7	2.8± 3.5*§
Akkermansia	5.5± 7.1	8.8± 9.9(*)	7.3± 10.6	7.6± 9.2	4.4± 5.3	21.6± 8**

Species level is not listed because no differences following intervention were found. Values are expressed as mean ± SD from 18 (PT, FO), or 9 (Fish intervention) participants. Analyses were measured before ("pre") and after ("post") intervention. Abbreviations: PT, Interventions with *Phaeodactylum tricornutum*; FO, Intervention with fish oil; Fish, Intervention with salmon. Statistics: * indicate differences to "pre", # indicate differences to baseline, § indicate differences to PTpost. (*) $p=$ 0.1, */§ $p < 0.05$, ** $p < 0.01$,
